# Association between Emotional Exhaustion and Tobacco Consumption in Teachers

**DOI:** 10.3390/ijerph19052606

**Published:** 2022-02-24

**Authors:** Alexis Portilla, María Fernanda Meza, Pablo A. Lizana

**Affiliations:** 1 Laboratory of Epidemiology and Morphological Sciences, Instituto de Biología, Pontificia Universidad Católica de Valparaíso, Valparaíso 2373223, Chile; alexis.portilla.s@mail.pucv.cl (A.P.); maria.meza.t@mail.pucv.cl (M.F.M.); 2 Programa de Magíster en Didáctica de las Ciencias Experimentales; Facultad de Ciencias, Pontificia Universidad Católica de Valparaíso, Valparaíso 2373223, Chile

**Keywords:** mental health, tobacco consumption, emotional exhaustion, teachers

## Abstract

Teachers have been reported as having high levels of emotional exhaustion (EE). It has also been observed that tobacco consumption (TC) is higher during stressful events. However, there is little evidence about the association between EE and TC among teachers. A total of *n* = 560 teachers took part in this study, where 71.79% (*n* = 402) were women. For data gathering, the EE dimension of the Maslach Inventory for teachers was used, along with a TC questionnaire and the sociodemographic data of the participants. A binary logistical regression model was used for statistical analysis. Regarding TC, over 30% of teachers declared that they smoked; 65% of the teachers presented medium-high EE and 31% of teachers presented high EE. Teachers who said they were smokers had a greater risk of presenting high EE (OR: 1.7, *p* < 0.05), along with younger teachers (≤44 years; OR: 2.1, *p* < 0.01). In addition, teachers with high EE also have a high risk of TC. The present study reports an association between TC and high EE category among teachers, regardless of gender. An important association is also observed between the under-45 age group and high EE. These results indicate that teachers should have psychological support and interventions aiding them with facing work stress and TC habits, especially for younger teachers.

## 1. Introduction

There is an important body of precedents indicating that teachers have high work burdens and stress, developing due to their workloads in educational establishments and continuing in their homes. This includes class planning, creating new teaching and learning strategies, preparing material for students, reviewing tests and homework, massive classrooms, classroom discipline problems, ongoing educational reforms, organizational conflicts, and lack of time [1,2,3]. All these practices are associated with stressors that can cause demotivation, job absenteeism, personnel turnover, and health problems such as chronic non-communicable diseases (e.g., obesity) that directly affect their quality of life [1,4,5,6,7]. In this sense, it has been observed that obesity among teachers represents a high prevalence [1,4,5] due to work overload, carrying out work even outside working hours, and not allowing leisure activities such as physical activity [1]. This context can generate physical and mental problems, such as emotional exhaustion (EE) [8].

### 1.1. Emotional Exhaustion in Teachers 

EE is a condition that develops very slowly due to various factors affecting daily life, referring to a lack of energy and emotional resources to face work obligations [3]. It has also been observed that EE is one of the strongest predictors of job performance decreases out of the three components of burnout syndrome [9]. In this sense, high EE scores make a greater contribution to overall burnout syndrome scores among teachers [10].

Studies about EE effects among teachers indicate that is associated with demotivation about teaching [11,12], doubts about capacity to effectively teach [13,14], and negative direct relations with students’ median grades [15] and with job satisfaction [16]. In Chile, it has been observed that EE prevalence was around 27% in teachers three decades ago [17,18], with important gender differences where women teachers showed significantly higher EE than men (30.3% versus 22.3%, respectively [17]). These studies showed high EE levels, which are also the highest by comparison with other Latin American countries. Thus, the report on teacher health conditions reported that Chilean teachers have the highest EE (42.6%) by contrast with Argentina (39.9%), Uruguay (29.2), Peru (12.7%), Mexico (12.75%), and Ecuador (12.3%) [2]. Subsequently, in 2017, it was reported that over 60% of teachers presented a medium-high EE level, with women showing higher rates [19]. Thus, it can be observed that studies have indicated a gradual increase in EE among Chilean teachers, and it is relevant to determine factors associated with EE. 

### 1.2. Tobacco Addiction 

High work demands generate a series of health consequences and habits among teachers. One of the habits reported among teachers to relieve stress and tension is tobacco consumption (TC) [20,21]. TC is a leading cause of death worldwide. According to the World Health Organization (WHO), it is the cause of over 8 million deaths annually, where 87% of deaths are due to direct consumption [22]. The WHO also mentions TC as a common risk for four non-transmissible diseases: cardiovascular diseases, chronic respiratory diseases, cancer, and diabetes, all of which are highly relevant, since these diseases are responsible for 70% of annual global deaths and 80% of all deaths in North and South America [22]. 

TC prevalence in North and South America is 17.4%, with a generally higher rate among men than women [23]. According to the information in this report, Chile is the country with the highest TC in the Americas Region, leading at 38.7% by comparison with other countries present in the report. TC among Chilean women is also at 35.1%, which is the highest TC figure by comparison with women from other countries in the Americas.

The teachers’ work health report indicates that Chilean teachers have high stress levels [18]. It is also important to highlight the current elevated rates of tobacco addiction (daily and occasional smoking) detected among women teachers, which are statistically higher than the rates for male teachers [18]. During the same year, the nationwide consumption rate was 42% [24]. These figures are concerning, since within the sample of teachers, TC prevalence is above the national levels, i.e., of the 42% of the population, 30.6% are education professionals [18,24]. TC differences have also been observed by age groups, where 57.1% are under 35 years old—the age group with the highest TC rate—while 29.8% are teachers over 45 years old [25].

### 1.3. Tobacco Addiction and Mental Health 

Increased TC prevalence and the higher number of diseases associated with mental health makes these factors into an important public health problem, significantly reducing quality of life (QoL) [26]. The extant relation between TC and mental health has become increasingly clear over time. In fact, there are studies suggesting a direct proportionality between these two conditions [27,28]. Evidence of increased smoking in people with mental health problems was also addressed by Plurphanswat et al. 2017; they addressed the causality between smoking and mental health using an exogenous source such as the state cigarette tax, where they reported that smoking is a cause of poor mental health [27]. In addition, a longitudinal study found a causal relationship between smoking and an increased risk of depression [29]. Specific links have also been observed with emotional status, reporting alterations in morbidity, physical activity, and sleep, which damage smokers’ life expectancy [30]. A study in England clearly showed that over 40% of tobacco consumers in that country had some type of mental illness [31]. 

Considering this background, and the paucity of information at the national and international levels, the objective of this study is to associate EE and TC among Chilean teachers. In particular, the aim is to analyze the levels of EE and how this symptomatology was affected by the participants’ sociodemographic characteristics. Differences in EE levels according to nutritional status and TC will also be analyzed. The findings of the following study could generate actions to improve teachers’ mental health.

We expect to observe high levels of EE among teachers. In addition, we anticipate that teachers who smoke are at high risk of having EE. Likewise, teachers with high EE also have a high risk of being smokers.

## 2. Materials and Methods

### 2.1. Participants

**Figure 1:**
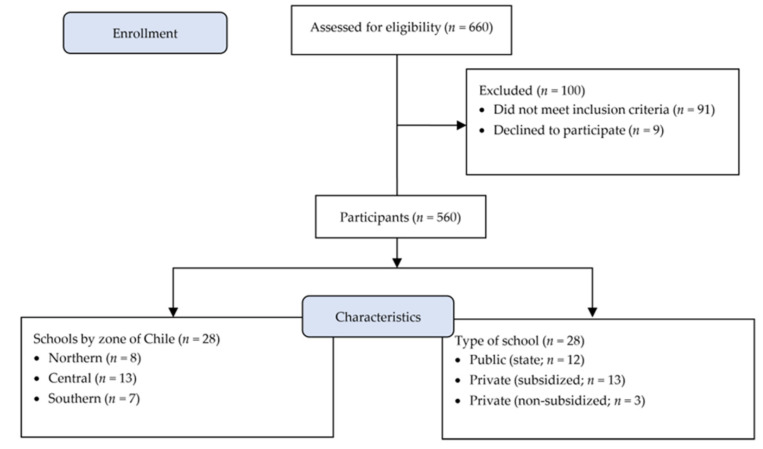
Flowchart for participants’ admissibility and characteristics.

This cross-sectional study was done among currently active teachers working in various types of schools (municipal/public, charter schools, and private schools—see Figure 1). The schools were randomly selected. These dependencies form part of the Chilean educational system, which predominate in the country, although it is in a process of change and transition. Regarding municipal public schools, these are the schools that receive contributions from the state for each student enrolled, while charter schools are establishments with shared funding between the state and the students’ parents. Finally, private schools are educational establishments sustained by monthly payments from parents and/or guardians. The target population was all teachers working in Chilean schools (*n* = 249865; MINEDUC, 2019 [32]). To calculate sample size, the variable with the highest variance was chose for this group in accordance with the published literature. The sample was determined with the variable of Chilean teachers’ EE (42.6%; [2]). The sample was calculated with 95% confidence and 5% error. Sample size also increased by 30% due to possible abandonment. The minimum sample was 537 participants. Sampling was conducted between 2018 and 2019. 

### 2.2. Instruments

#### 2.2.1. Sociodemographic Information

Teachers provided information about their age, gender, marital status, type of school, region, and city of residence within Chilean territory.

#### 2.2.2. Emotional Exhaustion

Teachers’ EE was evaluated via the subscale from the Maslach Inventory validated for Chilean teachers [33]. This includes nine items that were evaluated on a five-point Likert scale, ranging from 1 (not at all true) to 5 (completely true). One example item is “I feel fatigued when I get up in the morning and I have to face another day at school “. The scale showed good reliability (Cronbach’s alpha = 0.88).

#### 2.2.3. Tobacco Consumption 

Participants were considered as smokers according to the following criteria: daily smoker = person who has smoked at least one cigarette per day during the last 6 months; occasional smoker = person who has smoked less than one cigarette per day. Teachers who responded affirmatively to any of the questions were classified as “smokers”, and those who responded negatively were classified as “nonsmokers”.

#### 2.2.4. Anthropometry and Nutritional Status 

Anthropometric data collection was done in a time span no greater than 10 min by participants. The following points were considered for participants: full name, gender, date of birth, age in years, months and days, size at maximum inspiration, and weight with minimum clothing. Body mass index (BMI) was determined according to the following categories: underweight (BMI < 18.5 kg/m^2^), normal (18.5 kg/m^2^ ≤ BMI < 25 kg/m^2^), overweight (25 kg/m^2^ ≤ BMI < 30 kg/m^2^), and obese (BMI ≥ 30 kg/m^2^).

#### 2.2.5. Bioethical Aspects 

Prior to data collection, each participant had to read and sign an informed consent sheet where they were invited to voluntary and totally confidential participation in the study, which involved no remuneration, compensation, or conflict of interests with the researchers. In this sense, this study fulfills all the ethical requirements of the Helsinki Declaration and was approved by the Bioethics Committee at the Pontificia Universidad Católica de Valparaíso (#BIOEPUCV-H 160-2017).

#### 2.2.6. Statistical Analysis 

Data were analyzed with STATA version 16 software (StataCorp. Stata Statistical Software, College Station, TX: StataCorp. LP, USA). The internal consistency of the EE scale was evaluated with the Cronbach alpha coefficient where a value of 0.7 was considered satisfactory [34]. The sociodemographic variables’ associations were evaluated between each gender category via the chi-square test. Age was categorized according to the cutoff scores on the National Health Survey 2009–2010 (<45 years and ≥45 years) from the Chilean Ministry of Health [35]. The EE scores were categorized as follows: the scores of teachers from the 1st to the 33rd percentile (bottom third) will correspond to the low level of the EE dimension. Likewise, the scores of participants from the 34th to the 66th percentile (middle third) will correspond to a medium level of the EE dimension. Finally, the scores of subjects located in the top third will correspond to a high level of the EE dimension. The EE categories (low, medium, high) were compared with sociodemographic variables, BMI and tobacco addiction via the chi-square test, and any associations that turned out to be significant were incorporated into the multivariant model. Finally, two logistic regression models were performed. First, a regression model was performed with the category variable EE-high as the dependent variable to assess the association with TC. Second, to assess whether those teachers with high EE are also more tending to TC, TC was assessed as the dependent and independent variable. Both regression models were adjusted for the covariates of gender and age. The goodness of fit for each logistical regression model was demonstrated with the Hosmer–Lemeshow test.

## 3. Results

There were 660 initial participants in the study. Of these, 91 did not complete some of the surveys and had missing data, while nine abandoned the study. In total, we analyzed 560 teachers from 28 urban schools in three Chilean regions from different parts of the country, namely: Arica and Parinacota Region (northern zone); Valparaíso Region (central zone); and Araucanía Region (southern zone).

Table 1 shows the sociodemographic characteristics of the overall sample studied broken down by gender (male and female). A significant association appears between gender and marital status (*p* < 0.018) with a higher prevalence of cohabiting and married individuals among the men, reaching 48.7%. No associations were observed between gender and age, educational establishment type, BMI, and tobacco addiction.

Table 2 shows the participant sample categorized by EE level (low, medium, and high), where 28.2% were men and 71.8% were women, with no significant differences by EE category. However, a significant association was observed between EE category and age (*p* < 0.001) with a greater EE prevalence in the <45 group (77.2%). A significant association was also observed between EE categories and tobacco addiction (*p* < 0.05), where a higher rate of smokers was observed in the high EE category (39%). It is observed that as the EE category increases, the prevalence of TC and teachers under 45 years of age increases.

Table 3 shows the logistic regression model. Teachers who were self-declared smokers had a greater risk of presenting high EE (OR: 1.696, *p* < 0.05). With age, younger teachers (≤44 years) were associated with a significantly higher risk than teachers who were ≥45 years old of having high EE (OR: 2.124, *p* < 0.01). Finally, the gender variable saw no significant association within the model. In the goodness of fit test for the model (Hosmer–Lemeshow test), the value over 0.05 indicates that the model fits the data. Therefore, the model shows that independent of gender, younger teachers and tobacco users are those with the highest risk of presenting EE in the highest category. 

Table 4 shows the logistic regression model with smoking as the dependent variable. Teachers who have a high perception of EE have a higher risk of being smokers (OR: 1.696, *p* < 0.05). Therefore, the model shows that independent of gender and age, teachers with high EE also have a high risk of being tobacco users.

## 4. Discussion

### 4.1. Principal Results

The study presented a high number of overweight teachers, with the overweight and obese categories together adding up to 69% of the sample. For TC, over 30% of teachers declared that they smoked, with a higher rate among women. High category EE is more prevalent among people under 45 and teachers with smoking habits. In this sense, we can observe that there is a greater risk of presenting high EE among teachers under 45 and with smoking habits. There was also a high representation of female teachers (72%) above male teachers. The last aspect is consistent with international [2,36] and national studies [4,18,19,37,38], which note that the teaching profession has a strong female presence. 

### 4.2. Obesity among Teachers 

The results of this study indicate high proportions of overweight (43%) and obese teachers (23%). However, these are below the figures reported in the National Health Survey 2016–2017, which together (overweight, obese, and morbidly obese) reached 74.2% [39]. There is also a higher prevalence of teachers in the normal weight category (34%) than in the report of the NHS 2016–2017 (24.5%). These differences may be related to the traits of the profession, which requires moving around within the classroom, more standing time, various movements within the classroom including raising hands above shoulder height (blackboard work), and more [40,41]. However, these ergonomically disadvantageous movements can generate musculoskeletal disorders and affect teachers’ QoL [4]. The results of this study should also be taken with caution, since BMI is an index that cannot distinguish human body components, including muscle mass, bone mass, fatty mass, muscle content, and others [42]. In this sense, various studies among teachers have observed that BMI is undersized, and that evaluating obesity with methods that divide human body elements such as bioelectrical impedance to measure obesity by body fat percentage are a better measurement than BMI [4,5]. The results of this study also report obesity by BMI is higher among male teachers than females [5], but they differ from other studies that observed a higher obesity prevalence by BMI among females than males [4]. However, studies reported that teachers’ body fat percentages coincide in a higher rate among women [4,5].

### 4.3. Tobacco Consumption

This study found that the prevalence of smoking among teachers was 32%. This was a decrease by comparison with teachers’ tobacco consumption in 2003, which reached 35.9% [18]. However, Chilean teachers’ tobacco consumption by comparison with tobacco consumption in other countries is high. Erick et al. observed a TC prevalence of 3.2% among teachers in Botswana; Brazil presented higher TC values at 4.4%; and another study with teachers in Bangladesh presented a much greater rate, reaching 17% [43,44,45]. There is a trend in Chile for women to have higher cigarette consumption than men, with the National Teacher Health Report in 2003 and the subsequent NHS of 2017 reporting high cigarette consumption among women. These data are particularly concerning given that most teachers are female [4,5]. However, the present study does not show any statistical association between gender and tobacco consumption, which could indicate that other variables are influencing the high TC rates, such as teacher EE.

### 4.4. Emotional Exhaustion, Gender, Age, and Tobacco Consumption 

The results of the following study indicate that over one-third of teachers reported high EE (34.46%). High EE rates are consistent with the teacher health report (2005), indicating that Chilean teachers had the highest EE, followed by Argentina, Uruguay, Peru, Mexico, and finally Ecuador [2]. However, there is an observable increase in the high EE category compared with the report of Fuentes (2007) from Santiago de Chile in a sample of 112 teachers (32.1%). The results of the following study are concerning due to the high EE rate as well as the rise in frequency compared to previous literature. Apart from the negative effects generated in teacher performance, a negative relation has also been demonstrated between teacher EE and students’ median grades, standardized performance test results, school satisfaction, and perceived teacher support [15]. In this sense, teacher health is relevant for better work performance as well as having direct effects on student learning. 

Regarding EE and teachers’ gender, it could be observed that females presented a higher EE than their male peers (70% versus 30%, respectively in the high EE category). These results are consistent with those reported by various authors worldwide [36,46] as well as in Chile [1,19,38]. However, as with the group in Arvidsson et al. (2006), we could not detect associations by gender in the multivariant model associated with high EE. This result may be due to how in this study, females are highly represented (72%), matching with data from the Chilean Education Ministry where over 70% of teachers were women [32]. 

Concerning the participants’ age, it has been observed that people under 45 present a greater risk of high EE. This result is similar to that reported by De La Fuente (2007) [19], where people with the greatest EE in the high category were from the youngest age group (23–30 years) and the EE rate decreased in older age groups; the greatest rate of the low EE category arose in the oldest age group (55–62 years). These results match those of the following study where the over-45 age category had the strongest presence in the low EE category. In this sense, various studies have reported that younger teachers are more likely to have mental health impacts [5,38,47]. These results could be explained by the fact that older teachers present less stress due to resolving problems with greater independence and having more experience developing competence in their work routines [47]. These results are alarming, since it has been reported that around 40% of Chilean secondary school teachers leave the teaching profession during their first five years working in an educational institution, with their principal cited causes for leaving being work conditions and EE [48]. Therefore, these results indicate that tools need to be incorporated, which help face work stress elements, as well as improving teacher working conditions, especially for female teachers and teachers beginning their careers.

The bilateral association between high EE and TC among teachers (OR: 1.7) fits with studies indicating that people with mental problems consume more tobacco compared with tobacco non-consumers [49]. It has also been observed that tobacco addiction increases the number of bad mental health days, especially among individuals with more severe illnesses [27]. A cause–effect relation has also been observed between tobacco addiction and depression, indicating that greater cigarette consumption increases depression risk [31]. In addition, smoking behavior has been found to be associated with the first incidence of mental disorders, and smoking also doubles the likelihood of mental health problems compared to non-smokers [50]. In this regard, the mechanisms that may lead to changes in complex human behavior are still unclear. However, it is known that nicotine develops modifications in the nervous system that may affect cognition and aspects of emotional behavior [51,52]. In this sense, it has been observed that people tend to increase their TC in periods of increased stress [53,54]. Thus, in this study, we observed that as the EE category increases, so does the prevalence of TC (see Table 2), and teachers with high EE have a higher risk of TC (see Table 4). These findings are consistent with studies indicating that TC is higher in people with mental health problems [49,55]. In the case of teachers, it has been observed that they are under high levels of stress due to their high work overload [1,2,3]., which could be an important source of increased TC. In addition, the deterioration of teachers’ psychological health has been significantly correlated with smoking [56], and an increase in working hours has been associated with an increase in smoking among teachers [57]. In this respect, it has been observed that TC in teachers is mainly used for relaxation and stress relief [58] and as a coping strategy in work-related stress [59]. Together with the problems associated between TC and mental health, it is necessary to add an important body of evidence indicating TC as a cause of respiratory problems and cancer [50,60]. Therefore, we observe that TC can increase EE’s risk, but teachers with EE also increase the risk of increased consumption of EE. This phenomenon of stressed teachers and TC becomes a vicious circle that deteriorates teachers’ physical and mental health.

Finally, we observe that Chilean teachers present a high prevalence of obesity, TC, and EE. These conditions affect people’s physical and mental health but could also affect teachers’ performance and the learning outcomes of their students [15]. In this sense, teachers’ working conditions need to be reviewed, and it was already known that before the COVID-19 pandemic, the working conditions of teachers were stressful with little time for personal and family life with a substantial impact on their mental health. During the pandemic, the health of teachers worsened even more [38,61,62]. Therefore, it is essential to implement strategies to help teachers improve their physical and psychological health from teacher training, the first years in the profession, and develop vigilance throughout their working life.

### 4.5. Limitations

This study has some limitations that are relevant to describe for adequate interpretation of the results. The first limitation is about the nature of the study. As a cross-sectional study, it only allowed for a momentary observation of the participants, which can influence the response at the moment of the interview, and which also means that cause–effect relations cannot be done. The second limitation is that these data are from before the COVID-19 pandemic, which has seen a marked decrease in teacher QoL across the confinement periods, as well as a rise in emotional responses [38,61,62]. Therefore, high TC and EE rates could increase during critical periods. The third limitation is the sample. Although a representative sample of teachers was obtained, they do not necessarily represent the totality of teachers. However, the sample has the strength of covering three areas of Chile (north, center, and south), which aids with creating a more global vision of EE and TC problems among teachers. Finally, it is important to move forward with studies that describe the type and amount of tobacco that teachers consume, along with the reasons for TC.

## 5. Conclusions

This study reports an association between high category EE and TC among teachers, regardless of gender. An association was also observed between the under-45 age group and high EE. In addition, teachers with high EE also have a high risk of TC. Therefore, we suggest generating physical and mental health monitoring programs for teachers, as well as work organization evaluations to guarantee better QoL for teachers. Health policies that help reduce TC could also reduce mental health problems [27]. Teaching is a job that requires major mental labor; therefore, high EE and TC could affect job performance and students’ learning outcomes.

## Figures and Tables

**Table 1:** Sociodemographic characteristics of participants.

Characteristics	Total sample (*n* 560)		Male (*n* 158)		Female (*n* 402)		*p ^a^*
	*n*	%	*n*	%	*n*	%	
Age (years)							
<45	375	66.96	110	69.62	265	65.92	0.402
≥45	185	33.04	48	30.38	137	34.08	
Marital status							
Single	248	44.29	76	48.10	172	42.79	0.018
Married/partnered	265	47.32	77	48.73	188	46.77	
DWW	47	8.39	5	3.16	42	10.45	
Type of school							
Public (state)	187	33.39	44	27.85	143	35.57	0.084
Private (subsidized)	320	57.14	102	64.56	218	54.23	
Private (non-subsidized)	53	9.46	12	7.59	41	10.20	
Body Mass Index							
Eutrophic	189	33.75	43	27.22	146	36.32	0.099
Overweight	241	43.04	72	45.57	169	42.04	
Obese	130	23.21	43	27.22	87	21.64	
Tobacco Consumption							
Nonsmokers	381	68.04	112	70.89	269	66.92	0.365
Smokers	179	31.96	46	29.11	133	33.08	

DWW, Divorced/Widow/Widower; *p* < 0.05. ^a^ Chi-squared test.

**Table 2:** Demographic characteristics of the teachers associated with emotional exhaustion categories ^a^.

Characteristics	Low		Medium		High		*p ^b^*
	*n*	%	*n*	%	*n*	%	
Gender							
Male	49	26.92	51	27.57	58	30.05	0.775
Female	133	73.08	134	72.43	135	69.95	
Marital status							
Single	81	44.51	71	38.38	96	49.74	0.250
Married/partnered	84	46.15	97	52.43	84	43.52	
DWW	17	9.34	17	9.19	13	6.74	
Age (years)							
<45	111	60.99	115	62.16	149	77.2	0.001
≥45	71	39.01	70	37.84	44	22.8	
Type of school							
Public (state)	50	22.47	66	35.68	71	36.79	0.274
Private (subsidized)	116	63.74	100	54.05	104	53.89	
Private (non-subsidized)	16	8.79	19	10.27	18	9.33	
Body Mass Index							
Eutrophic	57	31.32	62	33.51	70	36.27	0.850
Overweight	79	43.41	82	44.32	80	41.45	
Obese	46	25.27	41	22.16	43	22.28	
Tobacco Consumption							
Nonsmokers	135	74.18	129	69.73	117	60.62	0.016
Smokers	47	25.82	56	30.27	76	39.38	

DWW, Divorced/Widow/Widower; *p* < 0.05. ^a^ EE scores were categorized into tertiles. ^b^ Chi-squared test.

**Table 3:** Logistical regressions for association of the emotional exhaustion high with tobacco consumption adjusted by gender and age.

Emotional Exhaustion High	OR	SE	95% CI		*p*
			*LL*	UL	
Tobacco Consumption (Smokers)	1.696	0.323	1.167	2.464	0.006
Gender (female)	0.872	0.174	0.589	1.290	0.494
Age (<45 años)	2.124	0.433	1.424	3.168	<0.001
Hosmer–Lemeshow test ^a^	0.948				

^a^ A value above 0.05 indicates that the model fits the data. OR, odds ratios; CI, confidence interval; LL, lower limit; UL, upper limit; SE, standard error.

**Table 4:** Logistical regressions for association of tobacco consumption with emotional exhaustion high adjusted by gender and age.

Tobacco Consumption (Smokers)	OR	SE	95% CI		*p*
			*LL*	UL	
Emotional exhaustion high	1.696	0.323	1.167	2.464	0.006
Gender (female)	1.224	0.253	0.817	1.835	0.327
Age (<45 años)	0.927	0.183	0.630	1.364	0.701
Hosmer–Lemeshow test ^a^	0.948				

^a^ A value above 0.05 indicates that the model fits the data. OR, odds ratios; CI, confidence interval; LL, lower limit; UL, upper limit; SE, standard error.

## Data Availability

The datasets generated and/or analyzed during the current study are available from the corresponding author on reasonable request.
